# Ethyl Acetate Fraction from *Hedyotis diffusa* plus *Scutellaria barbata* Exerts Anti-Breast Cancer Effect via miR-200c-PDE7B/PD-L1-AKT/MAPK Axis

**DOI:** 10.1155/2020/3587095

**Published:** 2020-08-19

**Authors:** Yue Yang, Ting Fang, Yi-Lan Cao, Ya-Xin Lv, Qing-Qi Chang, Dan-Dan Zhang

**Affiliations:** ^1^Institute of Interdisciplinary Integrative Medicine Research, Shanghai University of Traditional Chinese Medicine, Shanghai 201203, China; ^2^School of Pharmacy, Fujian University of Traditional Chinese Medicine, Fujian 350122, China; ^3^School of Pharmacy, Shanghai University of Traditional Chinese Medicine, Shanghai 201203, China

## Abstract

**Background:**

*Hedyotis diffusa* (HD) Willd. and *Scutellaria barbata* (SB) D. Don in different ratios have been frequently used to treat various cancers in clinical Traditional Chinese Medicine prescriptions. However, the optimal ratio, active fraction, and molecular mechanisms associated with the anti-breast cancer role of this herbal couplet have not been elaborated.

**Methods:**

To screen out the optimal ratio of this herbal couplet, we compare aqueous extracts of HD, SB, or HD plus SB in different weight ratios (HS11, HS12, HS21) for their anticancer effects on murine breast cancer 4T1 cells *in vitro* and *in vivo*. EA11, the ethyl acetate fraction from HS11 (the aqueous extract of the couplet at an equal weight ratio), is further assessed for its antiproliferative effect as well as the antitumorigenic impact with the aid of immunocompetent mice. Colony formation, flow cytometry, western blot, ELISA, and qRT-PCR are used to elucidate mechanisms underlying EA11-led effects.

**Results:**

HS11 presents the most potential suppression of 4T1 cell proliferation and tumor growth among these aqueous extracts. The comparison results show that EA11 is more effective than HS11 *in vitro* and *in vivo*. EA11 inhibits colony formation and induces apoptosis in a concentration-dependent manner. EA11 reduces the protein expressions of PDE7B, PD-L1, *β*-catenin, and cyclin D1 while elevating the concentration of cellular cAMP and miR-200c expression in 4T1 cells. Additionally, EA11 exerts its anticancer effect partially via the inactivation of MAPK and AKT signaling pathways.

**Conclusions:**

This study implicates that EA11 prevents breast tumor development by interfering with the miR-200c-PDE7B/PD-L1-AKT/MAPK axis. EA11 may represent a potential therapeutic candidate for breast cancer.

## 1. Introduction

Breast cancer is a common neoplasm globally with a high incidence in younger women nowadays [1]. Triple-negative breast cancer (TNBC) accounts for 15–20% of all kinds of breast cancer as the most challenging subtype to cure. TNBCs are characterized by absence of estrogen receptor (ER), progesterone receptor (PR), and human epidermal growth factor receptor 2 (HER2). Moreover, the late-stage of TNBC is often accompanied by metastasis to bone, liver, and brain. Although surgery, radiation, and chemotherapy may improve the survival of TNBC patients, the mortality rate of recurrent patients is still high. Therefore, a novel therapeutic strategy is desirable for TNBCs. Cancer immunotherapy has demonstrated promising outcomes in the treatment of melanoma and lung cancer [2]. The programmed death ligand-1 (PD-L1)/programmed cell death receptor (PD-1) pathway plays a crucial role in immunotherapy. Notably, the binding within PD-L1 in tumor and PD-1 on the cell surface of T cells to inhibit cytotoxic T cell responses leads to immune surveillance and tumor development [3, 4]. Recently, immunological checkpoint inhibitors have shown the efficiency of various cancers [5]. Anti-PD-L1 antibodies including atezolizumab (MPDL3280A), durvalumab (MEDI4736), avelumab, and pembrolizumab have been approved by Food and Drug Administration for the treatment of melanoma, Hodgkin's lymphoma, urothelial carcinoma, metastatic head and neck squamous cell carcinoma, and lung cancer [6–9].

Cyclic nucleotide phosphodiesterases (PDEs) are a multigene enzyme family that catalyzes the hydrolysis of cAMP (cyclic adenosine monophosphate) and cGMP (cyclic guanosine monophosphate). Among them, cyclic nucleotide phosphodiesterase isoform 7B (PDE7B) can specifically hydrolyze cAMP and regulate the intracellular concentrations, signaling pathways, and downstream biological functions of cAMP [10]. The higher level of PDE7B occurs in most glioblastoma cases and harms survival. The interaction between glioblastoma and endothelial cells leads to elevated PDE7B expression, which promotes tumor growth and aggressiveness [11]. PDE7 inhibitors selectively induce apoptosis in chronic lymphocytic leukemia cells due to PDE7B overexpression [12]. Therefore, PDE7B may be a potential target for some cancers.

Most mammalian mRNAs can be regulated by microRNAs (small noncoding RNAs, miRNAs) primarily at the posttranscriptional level, leading to either transcriptional silencing or degradation. miRNAs are 18–22-nucleotide endogenous noncoding RNAs that affect tumorigenic processes including cell proliferation, apoptosis, differentiation, metastasis, and drug resistance. There are five members in the miR-200 family: miR-200a, miR-200b, miR-200c, miR-141, and miR-429 [13]. miR-200c is a multifunctional miRNA capable of regulating cell migration, proliferation, drug resistance, stemness, and metabolism disorders [14]. Members of the miR-200 family can target PD-L1 and ZEB1 to suppress the exhaustion of CD8+ T cells and tumor metastasis in lung cancer [15]. miR-200c depressed PD-L1 expression and restored CD8+ T cell function in HBV-mediated hepatocellular carcinoma [16]. In the previous study, miR-200c regulated the overexpression of PDE7B in TNBC cells and were critical for TNBC cell proliferation and tumor development by modulating cellular cAMP concentration [17]. Current studies have shown that *Hedyotis diffusa* Willd. (HD), an annual herb of the Rubiaceae family, exhibited numerous pharmacological activities [18–21]. Pharmacological evidence revealed that *Scutellaria barbata* (SB) D. Don also displayed anticancer, anti-inflammation, and immunity-enhancing effects. The anticancer effects of HD and SB are most likely to be mediated through the inhibition of cell proliferation, induction of cell apoptosis, and regulation of immunity [22–25]. HD and SB were always used with dosage combination at 1 : 2, 2 : 1, or 1 : 1 in clinical practice. *Hedyotis diffusa* and *Scutellaria barbata* (HDSB) are the core drug pair for the treatment of breast cancer and bladder cancer by data mining from clinical drug databases [26, 27]. Our previous study showed that ethyl acetate fraction from the aqueous extract of the HDSB at equal weight ratio (EA11) has the most potent anti-inflammatory effect [28]. Given the close relationship between chronic inflammation and tumor, the objective of this study is to clarify the optimal ratio and active fraction from HDSB and to define the related antitumor mechanisms.

## 2. Materials and Methods

### 2.1. Cell Line and Culture Medium

The murine triple-negative breast cancer cell line 4T1 was purchased from the American Type Culture Collection (ATCC, Rockville, MD, USA). The cells were cultured in Dulbecco's Modified Eagle Medium (DMEM) (Gibco, California, USA) containing 10% FBS (Gibco, California, USA) in a 5% CO_2_ incubator (Thermo, Waltham, USA) at 37°C constant temperature.

### 2.2. Herbal Extract Preparation Procedure

These herbal aqueous extracts were prepared. First, the dried powders of HD and SB in different weight ratios or alone (HS11 : 450 mg each; HS21: HD-600 mg, SB-300 mg; HS12: HD-300 mg, SB-600 mg; HD : 900 mg; SB : 900 mg) were mixed and boiled for 2 H. These supernatants were collected, filtrated, and concentrated separately. These herbal extracts were stored at −20°C for further use. The source and identification of HD and SB, and the preparation and quality control of EA11 were described in the previous study [28].

### 2.3. Cell Viability Assay

Cell viability was measured using MTT assay [28].

### 2.4. Immunocompetent Mice Bearing 4T1 Tumor

All procedures performed in animal studies were approved by the Institutional Animal Care and Use Committee of Shanghai University of Traditional Chinese Medicine. The Female Balb/c mice (6 weeks old) were purchased from Shanghai SLAC Laboratory Animal Co., Ltd., and maintained in a pathogen-free environment. 4T1 cells (5*∗*10*E*5/100 *μ*l) were injected into second mammary fat pads of mice. One week after cell injection, mice were randomly assigned to the following groups (6 mice/group): Model (vehicle, ig), HD, SB, HS11, HS12, HS21 (25 g crude herb/kg/d, ig), and EA11 (50 mg/kg/d, ig). Mice were sacrificed after two weeks. Tumors were stripped, weighed, snap-frozen in liquid nitrogen, and stored at −80°C for subsequent experiments.

### 2.5. Colony Formation Assay

Cells were seeded in a 6-well plate for adherence overnight. The cells were treated with EA11 (25, 50 *μ*g/ml) for 24 h in serum-free DMEM and replaced with 10% FBS DMEM for six days. Cells were fixed with methanol and stained with 0.5% crystal violet (Genview, New Jersey, USA), and then the number of colonies was calculated.

### 2.6. Flow Cytometry

Apoptosis assay was performed using the PE/7-AAD Apoptosis Detection Kit (BD Biosciences, San Diego, CA, USA) on FACSCalibur according to the instructions. ModFit LT 3.0 software was used to calculate the percentage of apoptotic cells.

### 2.7. Western Blotting Analysis

4T1 cells were treated with/without EA11 for 24 h, and cell lysates were collected using RIPA buffer (Beyotime Technology, Jiangsu, China) and mixed with phosphatase inhibitor (Roche, Basel, Switzerland). The concentrations of protein were determined by BCA Protein Assay Kit (Beyotime Technology, Jiangsu, China). Proteins were separated using 4–12% SDS-PAGE gels and transferred to PVDF membranes. The membranes were blocked and then incubated with the following antibodies: PD-L1 (Proteintech, 17952-1-AP, 1 : 1000), PDE7B (Abcam, ab170914, 1 : 1000), *β*-catenin (CST, 9582, 1 : 1000), cyclin D1 (Abcam, ab134175, 1 : 5000), GAPDH (Proteintech, 10494-1-AP, 1 : 10000), JNK (CST, 9252, 1 : 1000), p-JNK (CST, 9251, 1 : 1000), ERK1/2 (CST, 9102, 1 : 1000), p-ERK1/2 (CST, 9101, 1 : 1000), p38/MAPK (CST, 9212, 1 : 1000), p-p38/MAPK (CST, 9211, 1 : 1000), p-AKT (CST, 4060, 1 : 1000), and AKT (CST, 9272, 1 : 1000). Membranes were washed with PBS-Tween-20 buffer (PBS-T) and incubated with goat anti-rabbit IgG (Abcam, ab6721, 1 : 10000). Blots were developed using ECL detection reagent (Millipore, Bedford, MA, USA). Protein bands were analyzed using the Tanon Imaging System (Tanon, Shanghai, China), and these band densities were quantified using the Tanon Program.

### 2.8. TaqMan® MicroRNA Real-Time RT-PCR Assays

Total RNA from each group was extracted by Trizol reagent (Invitrogen). hsa-miR-200c and the housekeeping gene U6 were determined by miScript II RT Kit and miScript SYBR Green PCR Kit (ABI, Foster City, CA, USA) according to the manufacturer's protocol. Real-time PCR amplifications were performed on ABI 7500 Fast Instrument. hsa-miR-200c expression was normalized to the endogenous control U6.

### 2.9. Determination of cAMP

The content of cAMP was determined using Cyclic AMP Select ELISA Kit (Cayman Chemical Company, Ann Arbor, MI) according to the manufacturer's protocol. 4T1 cells were treated with/without EA11 for 30 min before the analysis of cAMP.

### 2.10. Statistical Analysis

Statistical tests were performed using SPSS 20.0 (SPSS, Inc., Chicago, IL, USA). All results values are presented as mean ± standard deviation (SD) from three independent experiments. Statistical analysis was performed by *t*-test or one-way ANOVA to determine the statistical significance.

## 3. Results

### 3.1. EA11 Exhibits a Potent Antitumor Effect In Vitro and In Vivo

**Figure 1:**
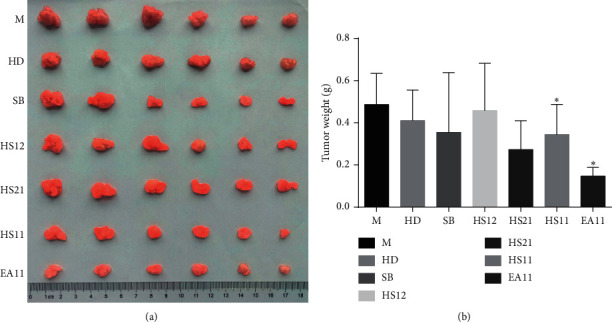
Effects on tumor growth of extracts from HDSB and EA11. M: model group; HD, SB, HS11, HS21, and HS11 at 25 g rude drug/kg/d; EA11: ethyl acetate fraction from HS11 at 50 mg/kg/d; data are presented as means ± standard deviation with three independent experiments. ^*∗*^*P* < 0.05 vs. M group.

Three weight ratios of HD and SB at 1 : 1, 2 : 1, or 1 : 2 are commonly used in clinical prescriptions to treat inflammatory diseases and cancers. We investigated the effect of aqueous extracts of HD, SB, HS11, HS12, HS21, and EA11 on 4T1 cell proliferation. MTT assay showed that HS11 exhibited the lowest IC_50_ at 226.93 *μ*g/mL among all tested aqueous extracts. EA11, the ethyl acetate fraction from HS11, displayed the most potent inhibitory effect at IC_50_ of 30.28 *μ*g/mL (Table 1). To further determine the impact of EA11 on tumor development, we injected 4T1 cells in female mice at second mammary fat pads. One week after cell injection, mice were administered with the vehicle control, HD, SB, HS11, HS12, HS21 (25 g crude drug/kg/d, equal to the clinical dosage), or EA11 (50 mg/kg/d) for two weeks. At the end of treatment, mice were sacrificed and tumors were excised. EA11 treatment group suppressed 66% of the tumor weight compared with the vehicle control group (Figure 1). These results showed that EA11 is the most potent in terms of suppressing tumor growth among all tested extracts.

### 3.2. EA11 Suppresses Colony Formation and Induces Apoptosis of 4T1 Cells

**Figure 2:**
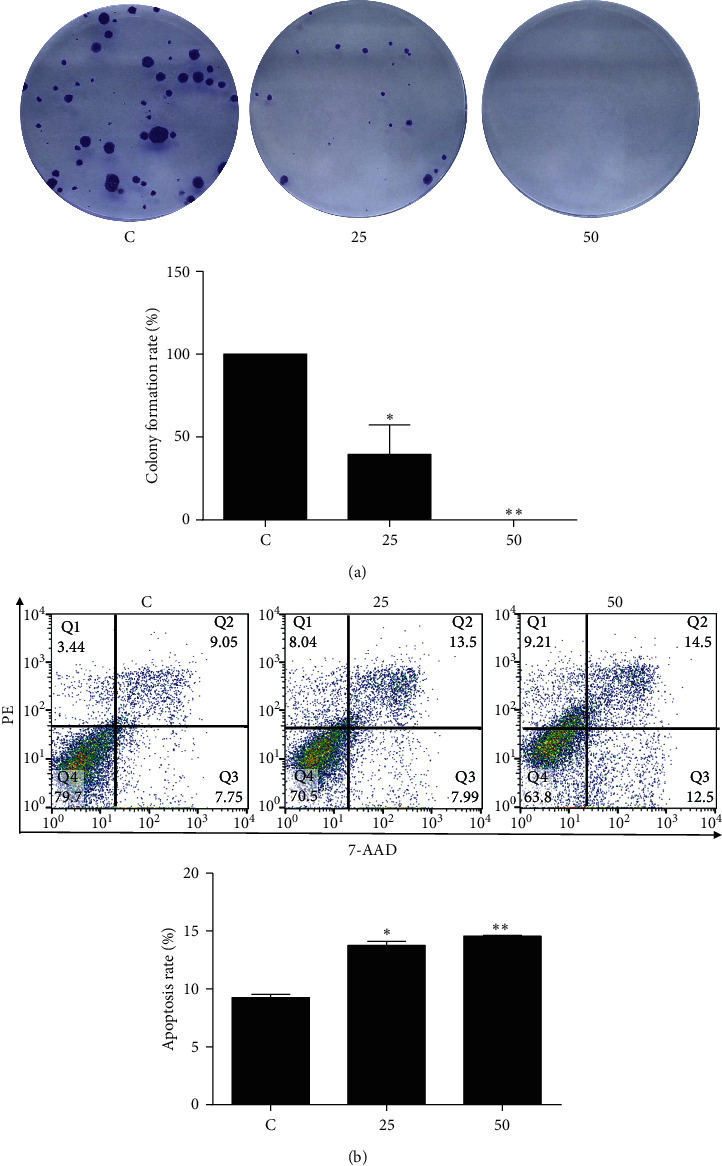
Colony formation inhibition and apoptosis induction of EA11. Following treatment of 4T1 cells with 25 or 50 *μ*g/ml of EA11, (a) colony formation assay and (b) cell apoptosis assay were carried out. ^*∗*^*P* < 0.05, ^*∗∗*^*P* < 0.01 vs. C group.

To further characterize EA11's antitumorigenic effect, we assessed the ability of EA11 to block the colony formation assay. Results showed that EA11 reduced the ability of 4T1 cells to form colonies in a concentration-dependent manner (Figure 2(a)). We also examined the effect of EA11 on apoptosis in 4T1 cells. The result of flow cytometry indicated that EA11 induced apoptosis in a concentration-dependent manner (Figure 2(b)).

### 3.3. EA11 Inhibits Protein Expressions of PD-L1, PDE-7B, *β*-Catenin, and Cyclin D1 in 4T1 Cells

**Figure 3:**
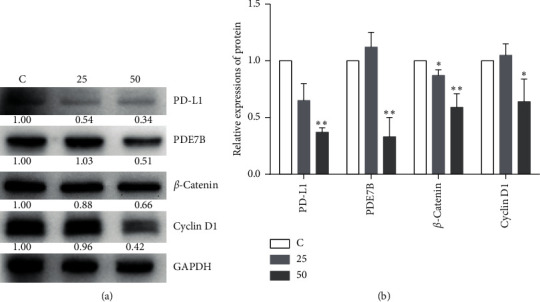
Analysis expressions of PDE7B, PD-L1, *β*-catenin and cyclin D1. Following the treatment of 4T1 cells with 25 or 50 *μ*g/ml of EA11, PDE7B, PD-L1, *β*-catenin and cyclin D1, proteins were assessed by western blot. Values are presented as mean ± SD from three experiments. ^*∗*^*P* < 0.05, ^*∗∗*^*P* < 0.01 vs. C group.

To define the molecular mechanisms associated with EA11-induced cell apoptosis, we analyzed the abundance of *β*-catenin and cyclin D1. As *β*-catenin and cyclin D1 are the critical components of the Wnt/*β*-catenin pathway and are known to be essential for cell proliferation and survival, we observed that EA11 decreased the expressions of *β*-catenin and cyclin D1 (Figure 3), suggesting that Wnt signaling pathway was involved in EA11-led action.

Overexpression of PD-L1 and PDE7B was observed in TNBCs, so we further determined whether their expressions were affected by EA11 treatment. Western blot with specific antibodies showed that EA11 treatment downregulated the expressions of PD-L1 and PDE7B (Figure 3). These results suggested that EA11 suppressed tumorigenesis by the blockage of the expressions of PDE7B and PD-L1.

### 3.4. EA11 Elevates the Level of Cellular cAMP

**Figure 4:**
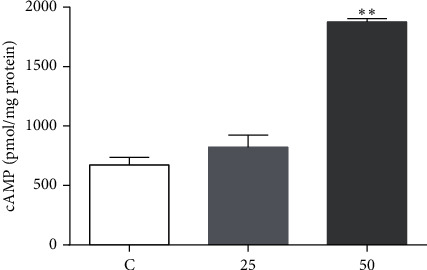
Determination of cAMP. Following the treatment of 4T1 cells with 25 or 50 *μ*g/ml EA11 for 30 min, the level of cAMP in each group was assessed by ELISA. ^*∗∗*^*P* < 0.01 vs. C group.

Inducing cellular cAMP level by inhibiting PDE7B expression or activity has been reported to trigger apoptosis in chronic lymphocytic leukemia and breast cancer [10, 17]. The observation that EA11 downregulated PDE7B expression prompted us to measure cellular cAMP concentration in EA11-treated 4T1 cells. The level of cellular cAMP was significantly elevated by EA11 treatment (Figure 4), suggesting that EA11 may induce apoptosis partially by increasing cellular cAMP concentration through the suppression of PDE7B.

### 3.5. Effects of EA11 on AKT/MAPK Signaling Pathways

**Figure 5:**
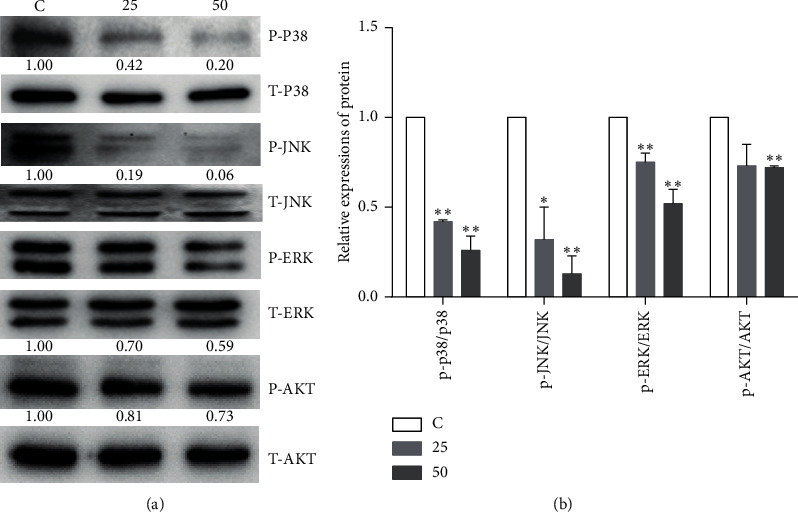
Effect of EA11 on the AKT and MAPK signaling pathways. p-ERK, ERK, p-JNK, JNK, p-p38, p38, p-AKT, and AKT protein expressions were assessed by western blot following treatment with 25 or 50 *μ*g/ml of EA11. Band intensity was determined using an imaging system. Band expression levels were calculated relative to the intensity of total JNK/ERK/p38/AKT protein. ^*∗*^*P* < 0.05, ^*∗∗*^*P* < 0.01 vs. C group.

PI3K/AKT and MAPK signaling pathways play vital roles in cell proliferation and survival. Both pathways are generally activated in TNBC cells [29, 30]. Western blot showed that EA11 inhibited the degree of AKT phosphorylation while not affecting the amount of total AKT. Similarly, we observed that the levels of phosphorylation of ERK, JNK, and P38 were all reduced upon EA11 treatment (Figure 5). These results suggested that EA11 may also exert its antitumorigenic effect by partially inhibiting AKT/MAPK signaling pathways.

### 3.6. Effect of EA11 on miR-200c Expression

**Figure 6:**
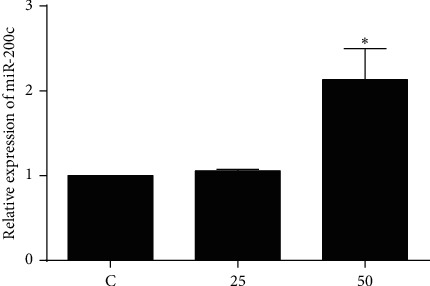
Effect of EA11 on miR-200c levels in murine 4T1 cells. 4T1 cells were incubated with the treatment of 25 and 50 *μ*g/ml EA11 for 24 h; ^*∗*^*P* < 0.05 vs. C group.

miRNAs directly or indirectly regulate AKT/MAPK signaling pathways. Our previous study showed that miR-200c-led inhibition of PDE7B diminished AKT activity to regulate TNBC cell proliferation and tumor growth [17]. Thus, we investigated whether miR-200c was associated with EA11-induced inactivation of AKT signal pathway. After the treatment with EA11, we observed that the level of miR-200c was significantly increased in 4T1 cells (Figure 6). These results indicated that EA11 induced miR-200c expression, leading to the inhibition of PDE7B expression and the subsequent inactivation of AKT signaling pathways.

## 4. Discussion

It is believed that tumors are the accumulation of heat and toxicity according to Traditional Chinese Medicine (TCM) theory. Therefore, the principle of clearing the heat and removing the toxins is one of the promising strategies for the treatment of cancer. *Hedyotis diffusa* and *Scutellaria barbata* have been widely used to treat various cancer types, and there are three weight ratios of this herb pair in prescriptions. However, the optimal weight ratio of HD plus SB in breast cancer treatment is not evaluated. In this study, we showed that HD plus SB at an equal weight ratio (HS11) exhibited a more potent antitumor effect than the other two weight ratios. EA11, the ethyl acetate fraction from HS11, displayed a more substantial impact on 4T1 cell proliferation and tumor growth than HS11 (Table 1). EA11 inhibited colony formation and induced cell apoptosis in a concentration-dependent manner (Figure 2). EA11 potently diminished the expressions of *β*-catenin and cyclin D1 (Figure 3); we reasoned the possibility that EA11 partially exerted its anticancer effect by interfering with Wnt signaling. MiR-200c regulates epithelial-mesenchymal transition (EMT) by blocking TGF-*β*, PI3K/AKT, Notch, VEGF, and NF-*κ*B signaling pathways [14]. miR-200c inhibited the expression of PDE7B, leading to an increased level of cellular cAMP and subsequent AKT inactivation and growth inhibition in TNBC cells [17]. In this study, EA11 increased the level of miR-200c, inhibited the expression of target gene PDE7B (Figures 3–6), elevated the concentration of cellular cAMP, and diminished the AKT activity (Figure 5). EA11 inhibited 4T1 proliferation and growth by the miR-200c-PDE7B-cAMP-AKT pathway.

Tumor cells can escape the surveillance from the body's immune system through a wide range of mechanisms including attraction of immunosuppressive cells, secretion of various cytokines and chemokines, and induction of immune checkpoint-mediated cosuppression signaling pathways. The expression of immunological checkpoint proteins in tumor cells results in the negative feedback of immune response and thereby promotes tumor development and metastasis [31]. Lack of miR-200c significantly increased the expressions of PD-L1 and MUC1 oncoprotein in acute myeloid leukemia, which showed that miR-200c was one of the critical negative regulators of PD-L1 [32]. Data analysis of 98 patients with HBV-associated hepatocellular carcinoma revealed that PD-L1 was negatively correlated with miR-200c [16] as well as that observed in clinical specimens of colon cancer [33]. In this study, EA11 robustly upregulated the expression of miR-200c to inhibit PD-L1 expression in 4T1 cells.

**Figure 7:**
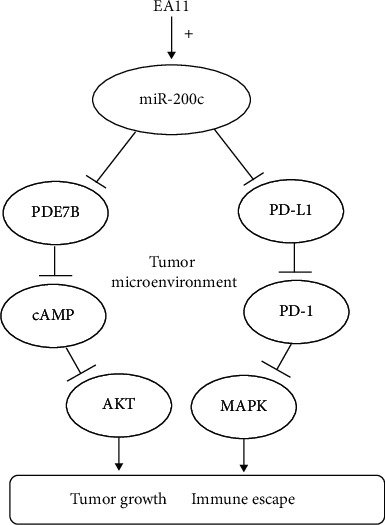
Potential molecular mechanisms by EA11. EA11 may induce miR-200c expression to regulate PDE7B and PD-L1 via AKT/MAPK signal pathways to suppress 4T1 proliferation and growth.

The MAPKs pathway is responsible for cell survival, proliferation, and differentiation including ERK1/2, JNK/SAPK, p38, and ERK5. Mutation-driven perturbations of MAPK pathways in breast tumors are linked to the negative regulation of immune response in breast cancer [34]. Combined Ras-MAPK pathway and PD-L1/PD-1 inhibition enhanced antitumor immune sensitivity in TNBC [35]. The data indicated that EA11 had inhibitory effects on phosphorylation of p38, JNK, and ERK/MAPK on 4T1 cells. Thus, inactivation of MAPKs pathway may boost the immune response by regulation of PD-L1 (Figure 5). In conclusion, our study demonstrated that EA11 could suppress breast tumor development by interfering with the miR-200c-PDE7B/PD-L1-AKT/MAPK axis (Figure 7). Based on these findings, EA11 can be developed as a chemopreventive agent for TNBC treatment.

## Figures and Tables

**Table 1:** Screening extracts from HDSB and EA11 on cell proliferation (*n* = 3).

Groups	Extracts/fractions	Dose (*μ*g/ml)	Inhibition (%)	IC_50_ (*μ*g/ml)	Yield (%)
Control	—	—	0.00		
HD	Water extract	250	43.75	336.36	13.48
		500	53.15		
		1000	81.68		
SB	Water extract	250	45.67	270.97	18.22
		500	59.51		
		1000	58.87		
HS12	Water extract	250	47.31	292.18	15.69
		500	56.53		
		1000	74.56		
HS21	Water extract	250	32.89	603.97	18.12
		500	43.83		
		1000	59.22		
HS11	Water extract	250	50.78	226.93	18.38
		500	68.52		
		1000	80.69		
EA11	Ethyl acetate fraction	62.5	80.41	30.28	0.26
		125	91.71		
		250	94.58		
		500	97.06		
		1000	97.14		
Tax		1 (*μ*M)	70.50		

Cell viability of 4T1 cells treated with extracts from HDSB and EA11 was determined by MTT assay. C: vehicle control; EA11: ethyl acetate fraction from HS11.

## Data Availability

The original data used to support the findings of this study are available from the corresponding author upon request.
